# A Review of Artificial Intelligence-Based Systems for Non-Invasive Glioblastoma Diagnosis

**DOI:** 10.3390/life15040643

**Published:** 2025-04-14

**Authors:** Kebin Contreras, Patricia E. Velez-Varela, Oscar Casanova-Carvajal, Angel Luis Alvarez, Ana Lorena Urbano-Bojorge

**Affiliations:** 1 Departamento de Biología, Facultad de Ciencias Naturales, Exactas y de la Educación FACNED, Universidad del Cauca, Popayán 190002, Colombia; 2 Centro de Tecnología Biomédica, Campus de Montegancedo, Universidad Politécnica de Madrid, 28040 Madrid, Spain; 3 Departamento de Eléctrica, Electrónica, Automática y Física Aplicada, Escuela Técnica Superior de Ingeniería y Diseño Industrial ETSIDI, Universidad Politécnica de Madrid, 28040 Madrid, Spain; 4 Escuela de Ingeniería de Fuenlabrada, Universidad Rey Juan Carlos, 28922 Madrid, Spain

**Keywords:** deep learning, machine learning, glioblastoma, tumors, magnetic resonance imaging, precision medicine

## Abstract

Background: Glioblastoma multiforme (GBM) is an aggressive brain tumor with a poor prognosis. Traditional diagnosis relies on invasive biopsies, which pose surgical risks. Advances in artificial intelligence (AI) and machine learning (ML) have improved non-invasive GBM diagnosis using magnetic resonance imaging (MRI), offering potential advantages in accuracy and efficiency. Objective: This review aims to identify the methodologies and technologies employed in AI-based GBM diagnostics. It further evaluates the performance of AI models using standard metrics, highlighting both their strengths and limitations. Methodology: In accordance with the preferred reporting items for systematic reviews and meta-analyses (PRISMA) guidelines, a systematic review was conducted across major academic databases. A total of 104 articles were identified in the initial search, and 15 studies were selected for final analysis after applying inclusion and exclusion criteria. Outcomes: The  included studies indicated  that the signal T1-weighted imaging (T1WI) is the most frequently used MRI modality in AI-based GBM diagnostics. Multimodal approaches integrating T1WI with diffusion-weighted imaging (DWI) and apparent diffusion coefficient (ADC) have demonstrated improved classification performance. Additionally, AI models have shown potential in surpassing conventional diagnostic methods, enabling automated tumor classification and enhancing prognostic predictions.

## 1. Introduction

Glioblastoma Multiforme (GBM) is a devastating and incurable brain tumor with a median overall survival of 15 months [1]. According to the World Health Organization (WHO), gliomas are classified from grade I to IV based on histological and molecular characteristics [2]. GBM, classified as a grade IV glioma, is the most invasive and treatment-resistant type. Gliomas account for 81% of malignant brain tumors, and GBM represents 60% of these cases [3,4]. Although treatment strategies exist, the 15-month survival rate for patients with GBM remains between 62% and 70% [5,6].

The clinical management of GBM involves safe surgical resection, which serves both diagnostic and therapeutic functions. It allows for histopathological confirmation through biopsy and reduces tumor volume through resection. Techniques include stereotactic needle biopsy, open craniotomy, and 5-ALA fluorescence-guided surgery [7]. However, while surgery is used for diagnosis and tumor reduction, it presents limitations that may influence subsequent treatment planning [8]. Surgical resection enables histopathological diagnosis and tumor removal. In contrast, biopsy is used in patients with deep-seated lesions, tumors in eloquent brain areas, or poor performance status [9].

Despite the limitations of stereotactic biopsies, tumor heterogeneity can be assessed by sampling multiple regions within the lesion. Multiregional sampling has revealed distinct clonal populations within a single tumor mass, highlighting the spatial genetic variability in GBM. These findings underscore the importance of sampling strategy when characterizing the molecular complexity of GBM in the context of individualized therapies [10]. Advances in neuroimaging, including multiparametric magnetic resonance imaging (MRI), have enabled the non-invasive assessment of tumor heterogeneity. Imaging biomarkers derived from quantitative MRI data, combined with texture and spatial analysis, can identify histologically distinct tumor subregions [11].

Following surgery, standard treatment includes chemoradiotherapy (CCRT) with temozolomide [12]. In patients with an unmethylated MGMT promoter or a Karnofsky performance status (KPS) below 70, radiotherapy (RT) alone may be used [13]. A diagnostic challenge after treatment is distinguishing tumor recurrence from radiation necrosis (RN), which develops within three years after RT. Both conditions appear similar on conventional MRI, showing contrast-enhancing lesions and surrounding edema, making interpretation and clinical decision-making difficult [14]. Since recurrence requires oncologic treatment while RN may be managed conservatively, precise diagnosis is necessary. This limitation has led to interest in MRI-based analysis as a complementary tool for diagnosis and treatment selection. Studies have identified biomarkers such as TRPM2 ion channels and TGF-β-related long non-coding RNAs (lncRNAs) as tools for non-invasive diagnosis and prognosis in GBM [15,16]. In addition, early detection remains essential for initiating appropriate treatment [17]. Although brain biopsy is the standard for diagnosis, it involves surgical risk, economic cost, waiting time, and may produce incomplete assessments of tumor heterogeneity [18,19].

To support non-invasive diagnosis, imaging modalities including MRI, perfusion imaging, diffusion tensor imaging, magnetic resonance spectroscopy, and molecular imaging have been applied [20]. Among these, MRI has been combined with artificial intelligence (AI) techniques for diagnostic modeling. Deep-learning models applied to MRI data classify brain pathologies, with studies reporting 98% precision in distinguishing GBM from cerebral metastases [21]. MRI supports tumor classification, treatment monitoring, and recurrence detection in clinical practice [3,22]. It also contributes to the non-invasive evaluation of brain disease [23]. Imaging-based diagnosis guides therapeutic decisions, as tumor subtypes vary in prognosis and require tailored approaches [24]. Radiomics enables the extraction of quantitative features from medical images, including shape, texture, and intensity, reflecting tumor characteristics [25]. AI models trained on imaging datasets support detection, classification, and clinical decisions [24,26,27].

This systematic review synthesizes developments in artificial intelligence-based systems for non-invasive glioblastoma diagnosis. It examines the performance of AI models applied to MRI data, including diagnostic accuracy, classification strategies, and prognostic potential. A specific emphasis is placed on identifying which MRI sequences provide the most informative features for AI-based classification of GBM. This work highlights the integration of radiomics with deep learning and presents an analysis of methodologies, validation approaches, and clinical implementation. The review provides researchers and clinicians with an overview of AI-based tools for GBM management.

## 2. Materials and Methods

This review was conducted in accordance with the guidelines outlined in “The PRISMA 2020 Statement: An Updated Guideline for Reporting Systematic Reviews” [28]. A systematic review of articles addressing the analysis of GBM using MRI and AI techniques was undertaken.

### 2.1. Protocol and Registration

The protocol was executed in adherence to the PRISMA-P guidelines. It was prospectively registered in the International Register of Systematic Reviews [29]. The initial registration was completed on 1 August 2024, with the most recent update recorded on 23 February 2025. The corresponding PROSPERO registration number is CRD42022368197.

### 2.2. Systematic Search

A systematic search was conducted on 1st August 2024, across the following databases: PubMed, Web of Science, Scopus, and Google Scholar, covering the period from 1 January 2018 to 4 February 2025. The search strategy was designed to ensure comprehensive coverage of relevant literature, incorporating key terms such as “Glioblastoma”, “Magnetic Resonance Imaging”, “Machine Learning”, and “Deep Learning”, while explicitly excluding terms such as “Surgical”, “Operative”, and “Treatment” to maintain a focus on GBM diagnosis prior to surgical intervention. A Boolean logic combination was applied using the NOT, AND, and OR operators, following a structured search string:

TITLE (glioblastoma OR gbm) AND TITLE (machine AND learning OR deep AND learning) AND ABS (magnetic AND resonance AND imag OR irm) AND NOT ABS (surgical OR operative OR treatment).

The following results were obtained:

- Scopus: 49 articles
- Web of Science: 31 articles
- PubMed: 23 articles
- Google Scholar: 1 article

The retrieved articles underwent a two-stage screening process based on predefined eligibility criteria, following the PRISMA 2020 Statement. The initial search identified a substantial number of publications related to cancer and AI. While several valuable scientific contributions were identified, others were deemed irrelevant to the specific objectives of this study. The review primarily focused on machine-learning approaches based on MRI for GBM diagnosis, leading to the establishment of well-defined inclusion and exclusion criteria for article selection.

Initially, titles and abstracts were screened to remove duplicates and exclude irrelevant studies. Subsequently, full-text articles were evaluated to determine their suitability based on the inclusion and exclusion criteria.

To enhance objectivity and reliability, the screening and selection process was conducted independently by two reviewers, with discrepancies resolved through discussion or by consulting a third reviewer in cases of disagreement. A PRISMA-compliant flow diagram was used to illustrate the selection process, documenting the number of records identified, screened, excluded, and included in the final synthesis. This approach ensured a systematic, transparent, and reproducible selection of studies, strengthening the validity of the review’s findings.

### 2.3. Inclusion Criteria

Inclusion criteria were defined to ensure that the systematic search remained strictly focused on the application of AI techniques for the diagnosis of GBM. The following inclusion criteria were applied in the review of the scientific literature:

- Scientific literature focused on artificial intelligence techniques.
- Scientific literature employed magnetic resonance imaging.
- Studies to address the diagnosis of GBM.
- Scientific literature exclusively of Quartils Q1 to Q2 from journals indexed in the Journal Citation Report (JCR).

### 2.4. Exclusion Criteria

Exclusion criteria were implemented to eliminate topics that did not align with the scope and objectives of this systematic review, including:

- Studies related to imaging techniques other than MRI.
- Evidence lacking sufficient methodological rigor or clinical relevance.
- Scientific literature in languages other than English.
- Editorials and other non-peer reviewed articles.
- Full-text not available.
- Studies conducted in animals.

### 2.5. Study Selection

**Figure 1:**
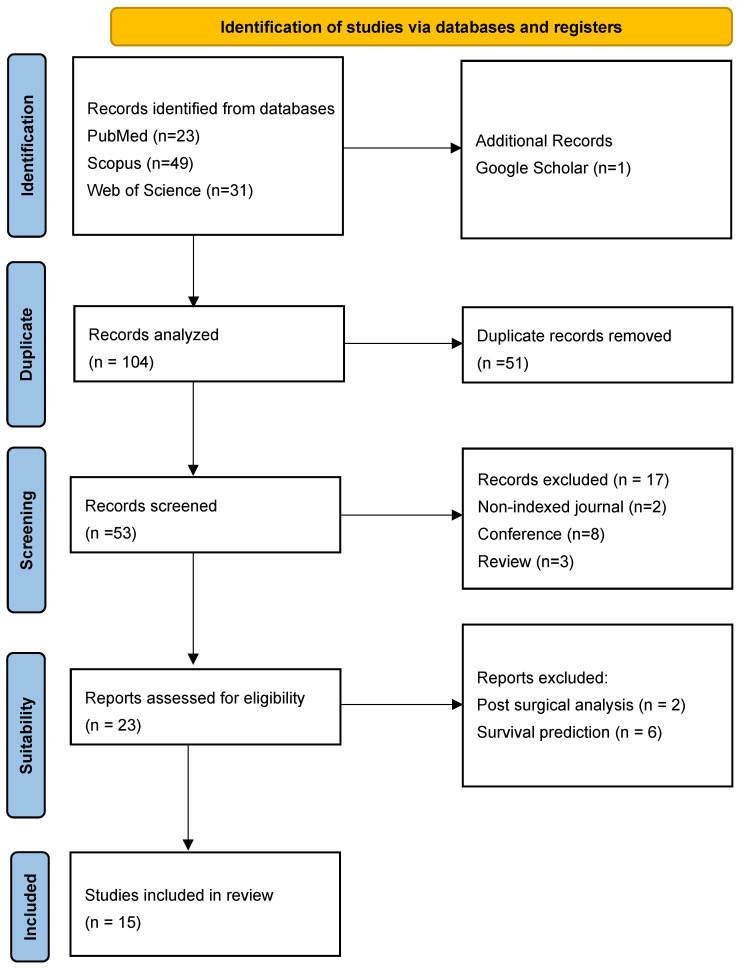
PRISMA methodology flowchart.

Articles retrieved through systematic search were subjected to filtering based on inclusion and exclusion criteria. Duplicate articles were removed and the abstract of each article was examined to ensure compliance with the proposed inclusion criteria. The articles selected for analysis in this systematic review were evaluated by the team members according to the following flow chart (Figure 1).

### 2.6. Data Extraction

All selected articles were manually extracted and synthesized in a predefined form, which included the following information: journals, quartiles, impact factor of the journal, year, authors, samples, main experiment, country, purpose of the study, data source, types of data, algorithms used, validation method and results (Table 1).

## 3. Results

The results were systematically structured into the following three sections according to the predominant clinical focus of each study: (3.1) tumor type and grade classification, (3.2) molecular biomarker prediction, and (3.3) comparison with clinical expert performance.

### 3.1. Tumor Type and Grade Classification

In [34], significant progress was demonstrated through the application of machine-learning techniques for cancer classification. Similarly, in [4], machine learning was employed for the semi-automatic classification of GBM, incorporating features derived from oxygen metabolism along with convolutional neural networks to assess patients with GBM or cerebral metastases. This approach achieved a significant improvement over traditional radiological evaluations, documenting an area under the curve (AUC) of 0.97 for differentiating these tumors. Furthermore, research referenced in [35] investigated the application of manual feature extraction and selection to enhance predictions of disease-free survival in various cancers, including GBM. These machine-learning models demonstrated in distinguishing GBM from brain metastases, achieving an AUC of 0.85% and a classification precision of 0.77%.

Beyond distinguishing GBM from cerebral metastases, machine-learning techniques have also been applied to differentiate GBM from other brain tumors. In [24], radiomic-based machine-learning technology was used to distinguish between GBM and anaplastic oligodendroglioma, achieving area under the curve values above 0.90%. Notably, models based on linear discriminant analysis (LDA) reached an AUC of up to 0.994 in the test group. Similarly, ref. [26] evaluated radiomic-based classifiers for differentiating GBM from primary central nervous system lymphoma (PCNSL), where the optimal LDA model achieved an AUC of 0.978, demonstrating robustness in cross-validation. These findings were further supported by [30], whose prediction model achieved an AUC of up to 0.977% by combining multiple magnetic resonance sequences, highlighting the potential of advanced techniques to optimize diagnostic precision in clinical settings.

### 3.2. Molecular Biomarker Prediction

The analysis carried out by [33] to predict the isocitrate dehydrogenase (IDH) mutation in patients with GBM using magnetic resonance imaging, employing a convolution neural network, showed a maximum accuracy of 77% in fluid-attenuated inversion recovery (FLAIR) images, highlighting the potential use of these techniques in the personalization of the treatment.

The study by [38] explored the prediction of early mortality in patients with GBM, using a naive Bayes classifier that achieved an AUC of 0.769% and a classification precision of 0. 80%, highlighting the ability of these tools for risk stratification. Currently, ref. [33] evaluated feature selection methods to differentiate between GBM and cerebral metastasis, achieving an accuracy of 0.77 and an AUC of 0.85 with the combination of least absolute shrinkage and selection operator (LASSO) and SVM, demonstrating the effectiveness of integrating techniques in radiomic analysis. Lastly, ref. [31] used the tree-based pipeline optimization tool (TPOT) to train predictive algorithms, reaching an AUC of 0.988 in the test group.

The work of [3] applied delta-radiomic characteristics derived from dynamic susceptibility contrast magnetic resonance imaging to differentiate between high- and low-grade gliomas, achieving an accuracy of 96% and an AUC of 0.94 in classification. Furthermore, ref. [25] used a cross-validation approach to analyze MRI images, achieving an average precision of 93.6% to classify necrosis and other critical characteristics of the tumor, emphasizing the utility of models based on random forests. Furthermore, the study by [30] demonstrated how combinations of different MRI sequences, such as the apparent diffusion coefficient (ADC), FLAIR and T1-CE, can achieve an AUC of up to 0.977, highlighting the precision of these models in differentiating between GBM and PCNSL.

**Figure 2:**
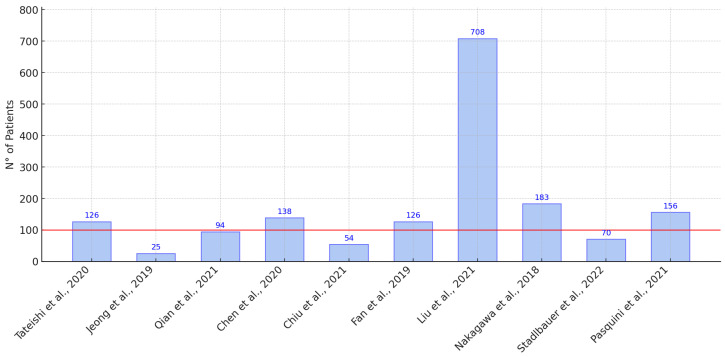
Distribution of sample sizes in the selected studies according to the references of the articles. The red line indicates the average sample size, with the outlier excluded (708 patients) [3,20,24,25,26,32,33,34,35,36].

As shown in Figure 2, the distribution of sample sizes between studies revealed a high degree of variability, with a notable outlier in 708 patients.

### 3.3. Comparison with Clinical Experts

On the other hand, ref. [36] developed a model using logistic regression and extreme gradient boosting (XGBoost) to differentiate between GBM and PCNSL, achieving a significantly high AUC of 0.98, which exceeded certified radiologists’ evaluations. Furthermore, the study by [20] used a support vector machine (SVM) to assess histogram and texture parameters in MRI, achieving an AUC of 0.92, comparable to the performance of expert radiologists, underscoring the applicability of these models in clinical practice.

**Figure 3:**
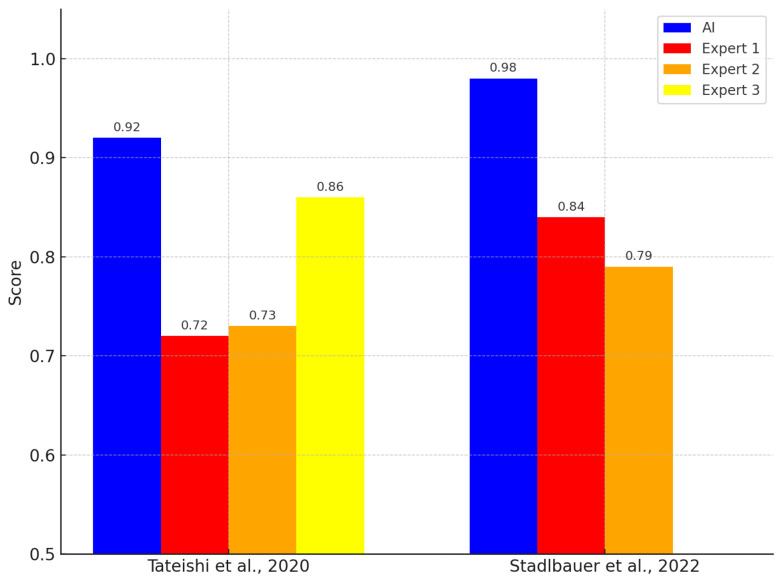
Comparative AUC scores between AI algorithms and multiple human experts in cancer analysis [20,34].

Finally, recent studies have shown that the application of machine-learning models has outperformed expert radiologists in terms of diagnostic precision. In the study by [20], the SVM model achieved an AUC of 0.92, compared to the AUC of experts, which was 0.72, 0.73, and 0.86. Similarly, ref. [36] reported that their XGBoost model achieved an AUC of 0.98, exceeding the AUC of expert radiologists, which was 0.84 and 0.79. These comparative outcomes are illustrated in Figure 3.

**Figure 4:**
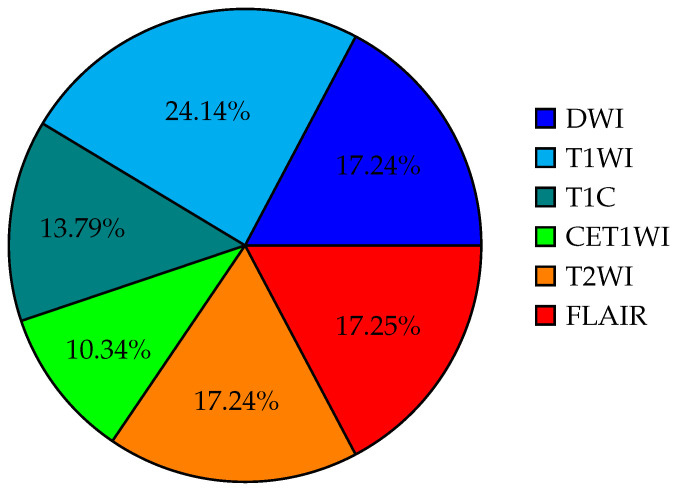
Percentage distribution of the use of different types of MRI signals in the selected studies.

Furthermore, the usage rates of various types of MRI signal in the reviewed studies are presented in Figure 4.

## 4. Discussion

 In line with the first group of studies, focused on tumor type and grade classification, machine-learning models achieved high diagnostic performance in differentiating glioblastoma from other intracranial tumors using MRI-based data.  High diagnostic performance was consistently reported; for example, an AUC of 0.975 with 92.1% sensitivity and 94.8% specificity was achieved when distinguishing GBM from brain metastases using radiomic characteristics of the T1C and FLAIR sequences [24]. Another study using convolutional neural networks reported an AUC of 0.98 when differentiating GBM from PCNSL, surpassing radiologists’ diagnostic accuracy (94% vs. 87%) [26]. Several other models also achieved AUCs above 0.97 [4,36]. In general, AI models matched or outperformed experienced radiologists in precision and consistency, reinforcing their potential for clinical application [20,34].

 Regarding molecular biomarker prediction and tumor characterization, models that incorporate CNNs and ensemble techniques such as XGBoost also produced excellent performance metrics.  ML models successfully classified GBM using radiomic features extracted from multiparametric MRI, allowing tumor characterization and patient stratification. CNNs and ensemble-learning techniques such as XGBoost produced AUCs ranging from 0.92 to 0.98 to distinguish GBM from brain metastases, PCNSL, and anaplastic oligodendroglioma [24,26,36]. These approaches support a clear early diagnosis and inform personalized treatment strategies. In particular, performance was more dependent on data quality and feature engineering than on dataset size, with studies employing dimensionality reduction techniques such as LASSO and PCA to improve generalizability [33,35].  In addition, some studies have extended these models toward non-invasive prediction of molecular features, such as IDH mutation status, aligning with the WHO classification of gliomas. These efforts demonstrate the growing role of AI in supporting molecular diagnosis through radiogenomic correlations. 

These results align with previous findings in neurooncology. Radiomic models consistently reported AUCs greater than 0.97 for the classification of gliomas [24,26]. For example, combining ADC, FLAIR, and T1C improved diagnostic accuracy to 94.3% [30], a strategy also adopted in [36]. Contrary to earlier meta-analyses, recent studies have demonstrated that small, well-curated datasets can yield high performance, with models trained on fewer than 100 patients achieving AUCs greater than 0.95 [3,35]. Key contributors to these outcomes include imaging quality, annotation consistency, and algorithm design.

 Studies that directly compared AI performance with clinical experts consistently reported better accuracy for AI-based approaches.  The implications are both clinical and methodological. Clinically, AI-based models can function as decision support tools in radiology, minimizing diagnostic delays and interobserver variability. For example, in [20], a CNN model reached the precision 94% for the diagnosis of GBM, outperforming radiologists (87%). Methodologically, the combination of multimodal imaging and radiomic characteristics underscores the need for standardized acquisition protocols and explainability tools to ensure interpretability. Deep-learning models applied to multiparametric MRI predicted the IDH mutation with an AUC of 0.88 [33], while early mortality prediction in GBM reached an AUC of 0.93 [38]. These findings support the role of AI in risk stratification and individualized treatment planning. The consistent performance gap between AI and human experts also highlights the need for regulatory oversight to ensure safe clinical deployment [20,36].

Although both studies [20,36] originated from the same institution, they differ in imaging protocols, patient cohorts, and analytical methods. Both used a 3T MAGNETOM Trio MRI system with a 12-channel phased array coil and included DWI, T2WI, and CE-T1WI sequences. However, ref. [36] also used DSC imaging to extract rCBV, which was not used in [20]. The patient cohorts addressed different diagnostic tasks: ref. [36] analyzed GBM versus PCNSL (*n* = 70), while ref. [20] focused on GBM versus MET (*n* = 126), with partially overlapping data periods. In terms of analysis, ref. [36] applied XGBoost with univariate logistic regression using features of multiple MRI modalities, including perfusion, while ref. [20] used an SVM trained in texture features of conventional sequences. Although both reported higher diagnostic accuracy for machine-learning models than radiologists, differences in sequences, features, and classifiers limit direct comparison. These distinctions suggest that the studies followed independent designs despite their shared origin.

This updated synthesis of AI applications in GBM diagnosis includes various ML algorithms and imaging modalities. CNNs, SVMs, logistic regression, and ensemble techniques such as XGBoost and TPOT were used [4,20,31,36]. Many models integrated hand-made and deep-learning features using multiparametric MRI sequences, including T1C, FLAIR, and ADC [35]. The evaluation of the model relied on cross-validation strategies, with most studies reporting AUCs greater than 0.90 [26,30]. Comparisons with expert radiologists further confirmed the strong performance of AI, with reported accuracies up to 94% [20].

Despite these strengths, several limitations were observed. The sample sizes varied substantially. For example, refs. [24,26] reported AUCs of 0.98 and 0.975 using fewer than 100 patients, while ref. [31] used a larger cohort (239 subjects) and reported a lower AUC of 0.86. These discrepancies suggest that data preprocessing, feature selection, and imaging quality may influence performance more than sample size. Furthermore, external validation was limited; only a minority of studies used independent test sets, restricting the assessment of model generalizability. Finally, standardization challenges persist, including variability in MRI protocols, feature definitions, and performance metrics, hindering reproducibility and clinical adoption. 

In line with these standardization issues, analysis of the included studies showed that MRI images were acquired at different institutions, contributing to the heterogeneity of the protocols. Eighty percent of the studies reported the type of scanner used, while the remaining 20% did not report this information. Of all the selected studies, the 80% used 3 Tesla (3T) scanners, while 20% also used 1.5T scanners for image acquisition. In particular, most of the scanners used were manufactured by Siemens Medical Systems (Erlangen, Germany), indicating a degree of manufacturer consistency, but not necessarily uniform acquisition protocols.

Looking ahead, future developments in AI-assisted diagnosis of GBM will likely focus on multimodal approaches that integrate imaging with genomic and clinical data. Explainable AI frameworks and federated learning are also emerging as promising strategies to enhance model transparency, protect data privacy, and facilitate regulatory compliance, paving the way for real-world clinical deployment. 

## 5. Conclusions

This systematic review identified three predominant clinical applications of machine-learning models for glioblastoma diagnosis using MRI: (1) tumor type and grade classification, (2) molecular biomarker prediction, and (3) comparison with clinical expert performance. AI-based models, particularly those using radiomic features and multiparametric MRI, demonstrated high diagnostic accuracy across all reviewed studies. In classification tasks, models achieved AUCs above 0.97 when distinguishing GBM from tumors such as PCNSL or metastases. In biomarker prediction, convolutional neural networks and ensemble models effectively inferred IDH mutation status and prognosis-related features. Additionally, several models outperformed radiologists in direct comparisons, supporting their value as clinical decision-support tools. 

These findings confirm the potential of AI to enhance non-invasive GBM diagnosis, support personalized treatment planning, and reduce interobserver variability. However, challenges remain regarding external validation, dataset standardization, and integration into clinical workflows. Future research should prioritize multicenter studies with standardized imaging protocols, explainable model outputs, and robust validation pipelines to ensure safe and effective clinical implementation. 

## Figures and Tables

**Table 1:** Individual results from the selected articles.

Reference Country Year	Description	Sample	Images							Model	Metric AUC	
		**Patient**	**DWI**	**T1WI**	**T1C**	**ADS**	**CET1WI**	**T2WI**	**FLAIR**		**AI**	**Exp. 1** **Exp. 2** **Exp. 3**
[20] JAPAN 2020	Initial machine-learning approach using multi-sequence MRI textures to differentiate glioblastoma from metastases.	126	X	X		X		X	X	SVM	0.92	0.72 0.73 0.86
[3] CHINA 2019	Classification of glioma using machine learning and delta-radiomic features from dynamic susceptibility contrast-enhanced MRI.	25	X	X				X	X	RF	0.94	— — —
[30] USA 2021	Radiomics-based differentiation between GBM and primary CNS lymphoma using multiple MRI sequences and machine-learning models.	94	X	X	X		X	X	X	ML RF, SVM,	0.97	— — —
[26] CHINA 2020	Comparative radiomics study for GBM and primary CNS lymphoma diagnosis using machine-learning classifiers.	138		X	X			X		LDA SVM LR	LDA: 0.978 SVM: 0.959 LR: 0.933	— — —
[25] TAIWAN 2021	Multiparametric MRI-based radiomics analysis for efficient tumor subregion classification of glioblastoma.	54	X	X	X			X		RF	Necrosis: 93.6 Solid part: 90.4 Peritumoral tissue: 95.8 Edema: 0.904	— — —
[24] CHINA 2019	Radiomics-based differentiation of GBM from anaplastic oligodendroglioma using advanced machine-learning techniques.	126			X					LDA, SVM	LDA + Dist. corr: 0.986 LDA + LASSO: 0.994 LDA + GBDT: 0.970 SVM + Dist. corr: 0.923 SVM + LASSO: 0.817	— — —
[31] CHINA 2022	Automated machine learning for image-based differentiation between GBM and metastasis.	708		X	X					TPOT	0.867	— — —
[32] CHINA 2021	Machine-learning analysis of MRI radiomics for gliosarcoma vs. glioblastoma classification.	183		X	X			X		SVM, AdaBoost, RF	0.85	— — —
[33] ITALY 2021	Deep-learning differentiation of IDH status in glioblastoma using multi-parametric MRI.	156	X	X	X		X	X	X	CNN	T1: 0.71 T2: 0.63 FLAIR: 0.74 MPRAGE: 0.62 ADC: 0.45	— — —
[4] USA 2019	Machine-learning semi-automation for classifying GBM, metastasis, and CNS lymphoma.	26			X	X		X	X	MLP, SVM	0.692	— — —
[34] AUSTRIA 2022	Differentiation of glioblastoma and brain metastases using oxygen metabolomic radiomics and deep learning.	133	X	X	X		X		X	1D-CNN	0.91	— — —
[35] CHINA 2021	Radiomic models for distinguishing GBM from brain metastasis using handcrafted and deep-learning features.	268	X	X	X		X	X	X	ML	0.97	— — —
[36] JAPAN 2018	Machine learning based on multi-parametric MRI to differentiate GBM from primary CNS lymphoma.	70	X		X			X	X	XGBoost	0.98	0.84 0.79 —
[37] USA 2021	Survival analysis in GBM using post-contrast MRI and multiple machine-learning models.	85	X	X			X	X		SVM XGBoost	0.811	— — —
[38] SPAIN 2021	Machine-learning analysis for predicting short-term survival after surgery in GBM cases.	203	X	X		X	X			RSF	0.769	— — —

Note: LDA (linear discriminant analysis), SVM (support vector machine), LR (logistic regression), LASSO (least absolute shrinkage and selection operator), RF (random forest),  T1C (T1 weighted contrast enhanced omaging),  CE-T1WI (contrast-enhanced T1-weighted imaging), and AUC (area under the curve).

## Abbreviations

The following abbreviations are used in this manuscript:

GBM	Glioblastoma multiforme
MRI	Magnetic resonance imaging
AI	Artificial intelligence
ML	Machine learning
DL	Deep learning
CNN	Convolutional neural network
AUC	Area under the curve
FLAIR	Fluid-attenuated inversion recovery
ADC	Apparent diffusion coefficient
T1WI	T1-weighted imaging
T2WI	T2-weighted imaging
T1C	T1-weighted contrast-enhanced imaging
DWI	Diffusion-weighted imaging
PCA	Principal component analysis
SVM	Support vector machine
RF	Random forest
LDA	Linear discriminant analysis
LR	Logistic regression
LASSO	Least absolute shrinkage and selection operator
GBDT	Gradient boosting decision tree
TPOT	Tree-based pipeline optimization tool
RSF	Random survival forest
PCNSL	Primary central nervous system lymphoma
BM	Brain metastases
MP	MaxPooling
WHO	World Health Organization
CE-T1WI	Contrast-enhanced T1-weighted imaging
